# Subdiffusion Supports Joining Of Correct Ends During Repair Of DNA Double-Strand Breaks

**DOI:** 10.1038/srep02511

**Published:** 2013-08-27

**Authors:** S. Girst, V. Hable, G. A. Drexler, C. Greubel, C. Siebenwirth, M. Haum, A. A. Friedl, G. Dollinger

**Affiliations:** 1 Angewandte Physik und Messtechnik LRT2, Universität der Bundeswehr München, 85577 Neubiberg, Germany; 2 Department of Radiation Oncology, University of Munich, 80336 Munich, Germany

## Abstract

The mobility of damaged chromatin regions in the nucleus may affect the probability of mis-repair. In this work, live-cell observation and distance tracking of GFP-tagged DNA damage response protein MDC1 was used to study the random-walk behaviour of chromatin domains containing radiation-induced DNA double-strand breaks (DSB). Our measurements indicate a subdiffusion-type random walk process with similar time dependence for isolated and clustered DSBs that were induced by 20 MeV proton or 43 MeV carbon ion micro-irradiation. As compared to normal diffusion, subdiffusion enhances the probability that both ends of a DSB meet, thus promoting high efficiency DNA repair. It also limits their probability of long-range movements and thus lowers the probability of mis-rejoining and chromosome aberrations.

DSB are a major threat for genomic integrity. However, some pathways for DSB repair can mis-rejoin DNA ends from different break sites, thus creating chromosome aberrations. The influence of initial distance between DSB sites on the probability of mis-rejoining is under debate, as well as the possibility of mechanisms bringing DSB sites into closer vicinity during damage processing^1^. Estimates on break interaction distances based on biophysical modelling vary between 100 nm and several μm^2^,^3^,^4^. Simulation models for DNA repair based on normal diffusion of ends tend to overestimate the fraction of unrejoined DSB after long repair times^5^.

Experimental data on the mobility of break sites in mammalian chromosomes are inconclusive. Mostly, they rely on labelling the chromatin region containing the DSB by visualizing specific chromatin modifications known to surround the breaks site, such as γ-H2AX, or proteins known to bind chromatin in the vicinity of break sites, such as MDC1. The labelled chromatin regions microscopically appear as ionizing radiation-induced foci (IRIF). Analyzing fixed cells, inferences on mobility are made by investigating the development of foci number and size over time, or their position with regard to the sites of damage induction^6^,^7^,^8^,^9^,^10^,^11^,^12^. In live-cell analysis, also the behaviour of individual foci over time can be observed and several authors measured trajectories in relation to the origin (single particle tracking^13^,^14^). In this case, thorough correction for translational and rotational movement of the whole nucleus, as well as its deformation, is paramount, since otherwise the measured mean squared displacements *MSD(Δt) = <r^2^(Δt)>* reflect movement of the nucleus rather than that of the foci within the nucleus.

A plot of MSD versus observation time allows determining if the particle observed moves via normal diffusion, in which case the MSD is linearly related to time and a diffusion coefficient *D* can be determined^15^,^16^. Assuming a linear dependence of MSD on time, recently a large diffusion coefficient was determined^13^ which was difficult to reconcile with the long-term stability of microscopic foci patterns after irradiation with ions in geometric patterns^9^. In addition, others reported for regions of undamaged chromatin that mobility is best described by subdiffusion (reviewed by^17^). In subdiffusion, MSD does not follow linear time dependence, but obeys 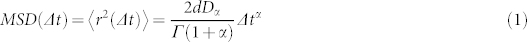
with *D_α_* being a generalized diffusion coefficient and α being the anomality parameter quantifying the deviation from normal diffusion. In the case of subdiffusion, *α* < 1, while in normal diffusion, *α* = 1. Γ represents the Gamma function and *d* is the dimension of the analyzed space.

To characterize the mobility of damaged chromatin regions, we chose to investigate distance changes between two IRIF over time rather than the MSD, using the standard deviation of the distance changes *σ^2^(Δl)*. While measuring distance changes has been introduced by others^18^,^19^ due to the obvious advantage over single-particle tracking in case of any translational and rotational movements of the nucleus, our method using the *σ^2^(Δl)* is even largely invariant in the case of additional changes of nucleus size.

## Results

### IRIF mobility follows a subdiffusion process

**Figure 1:**
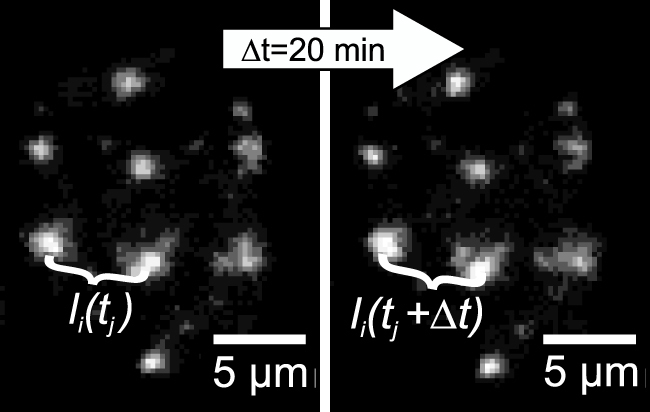
U2OS cell irradiated with carbon ions in a 5 × 5 μm^2^ matrix pattern, visible under the fluorescence microscope by GFP-tagging of DNA repair protein MDC1 which accumulates at the radiation induced double-strand breaks (“foci”). The distance *l_i_* between neighbouring foci is recorded at several time points *t_j_* for analyzing the distance changes *Δl_i_* over different time intervals *Δt*.

U2OS cells expressing GFP-tagged repair protein MDC1 were irradiated in a matrix pattern with single carbon (^12^C) ions per point. The distances *l_i_* of adjacent MDC1 foci pairs numbered by *i* were monitored for several hours post-irradiation (Fig. 1). The distance changes *Δl_i_(Δt)* during different time intervals *Δt* (i.e. *Δl_i_(Δt)* = *l_i_(t_j_ + Δt) − l_i_(t_j_)* for all time points *t_j_* of the time series) were determined.

**Figure 2:**
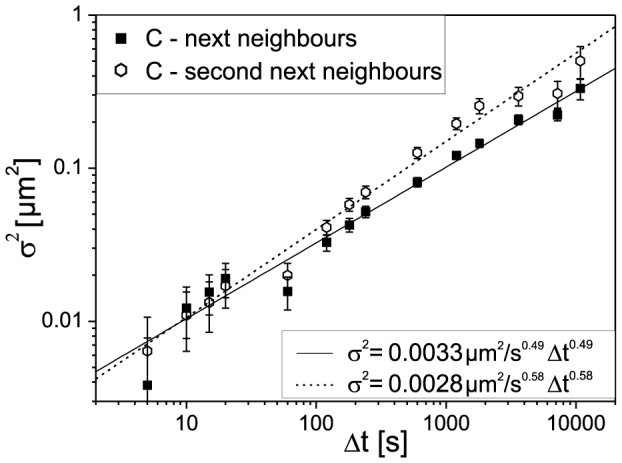
Double-logarithmic plot of the squared standard deviations *σ^2^* (±SEM) of the distance changes *Δl(Δt)* between neighbouring MDC1 foci (filled squares) and second-next neighbours (open circles) in the nuclei of cells irradiated with carbon ions (657 foci pairs from 58 cells, 30–60 time frames per sample). The data are fitted with the power-law function (equation (2)).

For each cell, we determined the standard deviation *σ^2^(Δl(Δt))* of the changes in foci distances *Δl_i_(Δt)* between neighbouring MDC1 foci as a measure for the underlying random walk process (see SI). Then the time-ensemble average of the “neighbouring” *σ^2^(Δt)* from all analyzed carbon irradiated cells was calculated for each *Δt* and plotted versus *Δt* in a double-logarithmic plot (Fig. 2, filled squares). A linear increase of log(*σ^2^*) with log(*Δt*) is evident, which demonstrates a power-law dependence of *σ^2^* on *Δt* (cf. Supplementary equation (S1)): 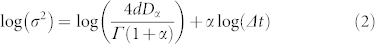
We obtain *α* = 0.49 ± 0.05 (SEM) and *D*_0.49_ = (3.7 ± 0.5) × 10^−4^ μm^2^/s^0.49^ for the next neighbour foci (*l* ≈ 5 μm) of the carbon irradiated cells (R^2^ = 0.97, reduced χ^2^ = 1.6). The value obtained for α significantly differs from *α* = 1 demonstrating that the mobility of the IRIFs follows a subdiffusion process^20^. Our data do not support models based on constrained diffusion^21^, since we did not see indications for a limit to the radius within which the IRIF can diffuse, in spite of the long time range (up to 10^4^ s) observed. In addition, the best least-square fits of constrained diffusion^19^ (R^2^ = 0.91, reduced χ^2^ = 5.0) or a combination of constrained diffusion and normal diffusion of the confinement region (R^2^ = 0.95, reduced χ^2^ = 2.8) did not fit the experimental data (Supplementary Fig. S2).

To examine the influence of the initial distances on the determined dynamics, the cells were additionally analyzed using the distances between second next foci neighbours (*l* ≈ 10 μm). As shown in Fig. 2 (open circles), the *σ^2^* analyzed from both data sets are very similar for *Δt* < 180 s, but increase faster in the case of second next neighbours at *Δt* > 180 s, hinting at non-corrected additional relative foci movements which we attribute to gross morphological deformations of cell nuclei. We determined *α* = 0.58 ± 0.05 (SEM) and *D*_0.58_ = (3.1 ± 0.5) × 10^−4^ μm^2^/s^0.58^.

Comparison between time-series recorded early (3–10 min) and late (80–100 min) after irradiation shows that the subdiffusion of the MDC1 foci is time-invariant, justifying the used method of ensemble- and time-averaging as it can be done for any ergodic process (Supplementary Fig. S3).

**Figure 3:**
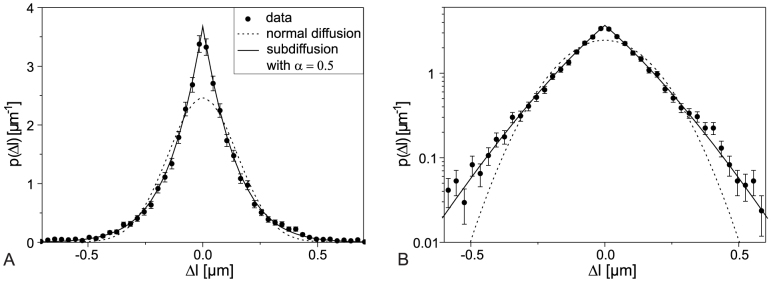
Histogram of distance changes (*Δl*(*Δt* = 60 s) between two neighbouring foci after carbon irradiation, fitted with the distribution function for normal and anomalous diffusion on linear (A) and logarithmic scale (B) (bin size = 0.03 μm, number of data points *N_total_* = 5656). SE-error bars are taken as 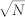 given through Poisson statistics.

The anomality of the diffusion of MDC1 foci can also be recognized in the histogram of distance changes *Δl_i_* for a single time interval *Δt* obtained for the carbon-irradiated samples (Fig. 3). Normal diffusion would lead to a Gaussian distribution of the probability density *p(Δl, Δt)* of the *Δl_i_(Δt)* values for initial distances much larger than the diffusional length^20^. The measured distribution function differs significantly from a Gaussian distribution, but is well fitted by the distribution function for subdiffusion (adapted from^20^) when using an anomalous diffusion exponent *α* = 0.5 (see Fig. 3, where *Δt* = 60 s) and applying a two dimensional random walk situation for distance analysis and thus substituting *x* → *Δl* and *K_α_* → *2dD_α_*: 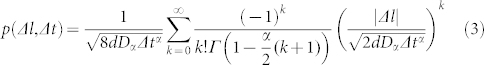
The differences to the Gaussian are visible both at very small distances, where a cusp-shaped histogram is obtained as predicted by the subdiffusion model (see plot on linear scale of Fig. 3a), as well as in the tails, where the distributions are significantly higher than predicted by a best Gaussian fit (log-plot of Fig. 3b). We conclude from our live-cell imaging and distance tracking over time periods between a few seconds and several hours after irradiation that IRIF do not diffuse normally but follow a subdiffusion process.

### LET dependence of apparent foci mobility

**Figure 4:**
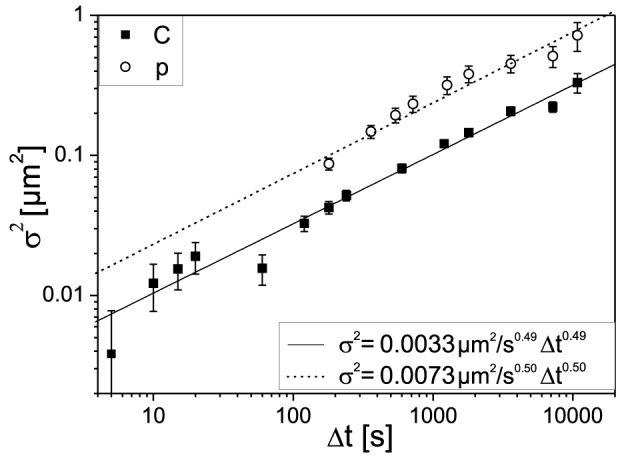
Double-logarithmic plot of the squared standard deviations *σ^2^* (±SEM) of the distance changes *Δl(Δt)* between neighbouring MDC1 foci in the nuclei of cells irradiated with carbon ions (filled squares, 657 foci pairs from 58 cells) and protons (open circles, 32 p per irradiation point, 99 foci pairs from 12 cells), each 30–60 time frames analyzed per sample. The data are fitted with the power-law function (eq. (2)).

To investigate whether IRIF diffusion depends on the density of damage sites along the irradiation track, the cells were also irradiated in a matrix of *Δx* = *Δy* = 6 μm with 32 protons (20 MeV at cell position, linear energy transfer LET = 2.6 keV/μm) per point. The number of protons was chosen to create on average 3 DSB per matrix point so that within the depth of view each detected IRIF contained on average only one DSB, while after carbon irradiation several DSB are expected to occur in each IRIF. As in the case of carbon-irradiated cells, a linear increase of log(*σ^2^*) with log(*Δt*) is evident (Fig. 4), demonstrating a power-law dependence of *σ^2^* on *Δt*. For comparison, also the data obtained after carbon ion irradiation are shown.

We determined *α* = 0.50 ± 0.05 (SEM) and *D*_0.50_ = (8.1 ± 1.8) × 10^−4^ μm^2^/s^0.50^ for the proton-irradiated cells, showing that the time dependence of foci after carbon and proton irradiation is very similar. As the anomality parameter α for proton and carbon irradiation (*α* = 0.50 ± 0.05, and *α* = 0.49 ± 0.05, respectively) is the same within the errors, the number of induced double-strand breaks within one focus does not seem to influence the degree of anomality of the foci diffusion. The absolute *σ^2^* values, however, differ by a factor 2.2. This difference may be attributed to the fact that the dynamics observed in proton irradiated cells is mainly that of an isolated, single DSB (number of DSB per IRIF *N_p_* ~ 1), whereas due to clustered DSB induction and poor axial microscopic resolution a number of *N_C_* > 1 foci contribute to each detectable IRIF after carbon irradiation. Thus, the diffusional behaviour observed is that of the centre of mass of all DSB in this IRIF, where contributions of individual DSB can neutralize each other. In a pure statistical approach we expect *σ^2^_p_/σ^2^_C_ = N_C_/N_p_* (see Supplementary Information “Random walk of the centre of mass of N DSB”). The measured ratio of 2.2 is approximately the number of DSB per micrometer as calculated in^8^ multiplied with the approximate depth of field of the used epifluorescence microscope.

**Figure 5:**
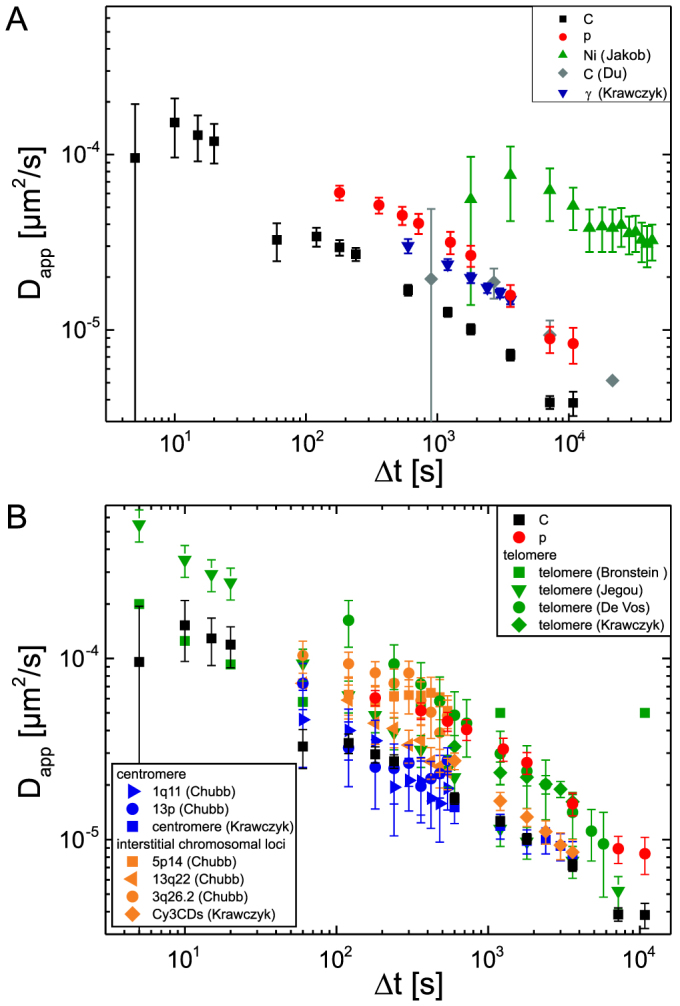
The apparent diffusion coefficient 
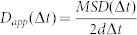
 of DSB induced by different ionizing radiation (A) and in comparison with that of centromeric, telomeric and interstitial chromosomal loci (B) (with standard errors). C and p are the data sets from this work (cf. Fig. 4). (A) “C (Du)” is the lateral diffusion data of NBS1-foci in BJ1-hTERT cells irradiated with C ions (~40 MeV) at SNAKE^11^ and “Ni (Jakob)” and “γ (Krawczyk)” are the diffusion data of GFP-tagged 53BP1-foci in U2OS cells irradiated with 6 MeV Ni ions^13^ and with γ^31^, respectively. (B) Telomere data extracted from Bronstein et al^25^., Jegou et al^19^., De Vos et al^33^. and Krawczyk et al^31^. Apparent diffusion coefficient of centromeric and interstitial chromosomal loci calculated from Chubb et al^18^. and Krawczyk et al^31^. *D_app_* is calculated using eq. (4) with corresponding dimension *d* and calculating MSD according to eq. (1) in case of distance measurements.

To experimentally verify this assumption, we rationalized that increasing the microscopic resolution would allow to differentiate between the IRIF of individual DSB. With small-angle irradiation it is possible to visualize with good lateral resolution how ion-induced IRIF form along the track^6^. Due to technical limitation, we were not able to perform live-cell analysis after small-angle carbon irradiation. We decided, therefore, to reinvestigate data obtained in earlier work (Du et al. 2011^11^) by NBS1 foci detection in carbon-irradiated cells fixed at various post-irradiation intervals. To allow a comparison of these data to our live-cell data, we introduce the “apparent diffusion coefficient” *D_app_*, which is defined as the ratio of the mean squared displacement *MSD(Δt)* (equation (1)) and the corresponding time *Δt*, corrected for the dimensionality *d* of the random walk: 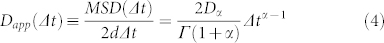
Our live-cell analysis has *d* = 2 due to the two-dimensional analysis of the centre of foci in the focal plane of the epifluorescence microscope, while the analysis in^11^ has been performed in one dimension. The apparent diffusion coefficient *D_app_* is equal to the average diffusion constant that would result in the same MSD if normal diffusion had taken place. It is evident (Fig. 5) that the mobility of IRIF induced by small-angle carbon irradiation compares well to the mobility determined in the present work for proton-induced IRIF.

## Discussion

In the present work, we demonstrate that radiation-induced MDC1 foci, which are assumed to reflect the chromatin region surrounding one DSB or several clustered DSB, exhibit a subdiffusive mobility in the time range 5 s ≤ *Δt* ≤ 10^4^ s, with *α* ≈ 0.5. Subdiffusive behaviour of chromatin regions has been described in yeast^22^, *E. coli* chromosomes^23^,^24^ and, at least in the time scale of up to 100 s, telomeric regions in mammalian U2OS cells^25^,^26^. Others, however, reported constrained diffusion of genomic loci (reviewed by^21^). Recently, Albert et al. (2012) proposed that genomic loci in general undergo subdiffusion at all time scales explored, and that this effect may be overlooked when plotting MSD on a linear scale, where constrained and subdiffusive mobility lead to very similar curves^17^.

The underlying mechanisms leading to subdiffusion have mainly been investigated in the context of anomalous mobility of macromolecules such as proteins and microinjected beads in nucleoplasm and cytoplasm (e.g.^27^,^28^) where subdiffusion appears to be a consequence of crowding-induced viscoelasticity^15^,^27^. Modelling results suggest that subdiffusive motion of chromosomal loci in *E. coli* is a consequence of both viscoelasticity of the nucleoplasm and the restrictions on monomer mobility caused by neighbouring monomers in a polymer^23^. Also transient immobilization by binding to obstacles may contribute to subdiffusive behaviour^29^,^30^.

The most pertinent characteristic of subdiffusion is that apparent mobility becomes slower at longer observation times. To detect subdiffusive behaviour it is therefore important to avoid overestimation of mobility at long observation intervals which may be caused by insufficient correction for mobility of the reference system, i.e. the nucleus in the case of chromosomal loci. We derived *D_app_* from a variety of data on mobility measurements of chromosomal loci available in the literature (Fig. 5a + b.). We note that those few mobility measurements which did not show a reduction of D_app_ with observation time were performed with single particle tracking (Fig. 5a “Ni (Jakob)”^13^, Fig. 5b “telomere (Bronstein)”^25^ at long *Δt*). In contrast, distance tracking as used in our work, intrinsically corrects for translational and rotational movements and, when using the standard deviation of distance changes as it is used here, even largely corrects for a change of the nucleus size. It is important to note that reduced apparent mobility at longer observation periods is not a consequence of altered conditions with increasing post-irradiation time (e.g. due to repair, or unphysiological conditions) since the mobility at short observation intervals is similar whether investigated within the first minutes after irradiation or after one hour (see Supplementary Fig. S3).

What are the consequences of subdiffusive mobility? As discussed by Weber (2010), “subdiffusive molecules take longer than expected to reach distant targets but they are also more likely to retrace local regions of space than a freely diffusing particle”^23^. Thus, the encountered subdiffusion predicts long-range movements of chromosomal loci and DSB to be rare, even at long time intervals. Figure 5a shows that our measurements for MDC1 and NBS1 foci^11^ mobility compare well with data on 53BP1 foci determined recently after γ irradiation^31^. Since contradictory data were published on whether IRIF can travel μm distances and coalesce to form repair centres^7^,^12^,^13^,^32^, it is interesting to compare IRIF mobility to that of undamaged chromatin regions. As evident from Fig. 5b, these data agree also well with a large set of published data on mobility of telomeric, centromeric, or interstitial chromosomal loci. In spite of the use of different methods and experimental systems, all available mobility data are surprisingly similar. Whether, as suggested by Krawczyk et al^31^., IRIF and telomeric loci exhibit generally a slightly enhanced mobility as compared to interstitial loci remains to be investigated in detail, also taking into account differences in foci substructures.

In any case, we conclude that any model invoking movements of foci over several μm within a few hours cannot be explained by typical mobility of chromosomal loci. Since the average distance of travel is small, the probability of joining wrong ends from DSB that initially were separated by several micrometers is low. For example, with typical values for *D_app_* smaller than 10^−5^ μm^2^/s at *Δt* = 3 h (Fig. 5b), the average sampled volume has a radius of 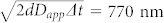 (*d* = 3) which is less than 0.5% of the total volume of the cell nucleus of about 700 μm^3^.

Our study investigated the mobility of IRIF surrounding break sites rather than the mobility of individual DNA ends. Little is known on the extent of tethering of both ends of a DSB site, resulting either from the original chromatin structure or from fast DSB detection and response mechanisms^32^. There must be a certain probability of separation between ends, since otherwise mis-rejoining and generation of aberrations would not occur. For our considerations, we assume that the single ends underlie subdiffusion of similar speed as the DSB site as a whole. If there was independent movement of both single ends at considerably faster velocity, the monitored IRIF would be expected to frequently dissociate into two foci, which was not seen, although rare dissociation events cannot be excluded from the limited statistics of the experiment. Furthermore, the DNA ends are still connected to the chromatin, which is found to follow subdiffusion also in the case of telomeric ends, with a mobility that agrees well with the IRIF mobility (cf. Fig. 5b). Computer simulations demonstrated that subdiffusion enhances the probability of finding nearby targets as compared to normal diffusion for initial separations *R* smaller than the so-called crossover radius *R_c_*, thus facilitating cellular processes such as protein complex formation with high probability^16^. A target size a = 2 nm to find a broken DNA end approximated by the diameter of the size of the DNA end, a nucleoplasmic viscosity of 2 × 10^−3^ Pa·s at 37°C and an anomalous diffusion coefficient *α* = 0.5 lead to a search time-dependent crossover radius *R_c_* [μm] ≈ 0.5 × *t_search_*^1/4^ (with *t_search_* in [s]) of more than 5 μm for search times of 1–3 hours (calculated according to^16^). The radius may be even enhanced by a factor 2^1/4^ = 1.2 because both ends of a DSB are subject to subdiffusion. Provided that the distance of the two ends of a DSB that had separated from each other after damage induction is smaller than *R_c_*, the probability that the ends encounter again would thus be larger in the case of subdiffusion than for normal diffusion. This means that subdiffusion supports the rejoining of ends during repair of DNA double-strand breaks. By labelling both sides of an enzyme-induced break site with different fluorescent molecules, Soutoglou et al. 2007 demonstrated typical separation distances in the range of *R* = 100 nm^32^, i.e. much smaller than the crossover radius *R_c_* of more than 5 μm estimated here for several hours of search/repair time. Thus, the rejoining probability (within a few hours after irradiation) can be estimated to be larger than 90% for subdiffusion (using the model of^16^), while normal diffusion would only rejoin broken ends with initial separation *R* ~ 100 nm with maximum 2% probability (*P(R) = a/R*^16^).

When there are more than one DSB within the initial separation distance R of the two ends of a single DSB, the probability to connect two ends would be the same for all DNA ends, thus resulting in a large fraction of translocations. Note that the ratio of correctly rejoined ends and translocations would be the same under subdiffusion or normal diffusion random walk, but many more single ends would be left in case of a normal diffusion. In the case that two DSB are created at a distance larger than R, however, the ratio of correctly rejoined ends to translocations increases more for subdiffusion than for normal diffusion.

In conclusion, we propose that the subdiffusional behaviour of DSB sites guarantees a high probability of rejoining the ends of an individual DSB while reducing the probability of chromosomal aberrations produced by rejoining the ends of different DSB or of leaving a large fraction of DNA ends unrepaired.

## Methods

### Cell culture and irradiation

Generation of a stable transfectant (clone F1) of human osteosarcoma cells (U2OS) with pEGFP-MDC1^34^ was seeded into custom-made cell containers^35^ in HEPES-buffered, phenol red-free medium supplemented with 0.25 mM Trolox at 37°C and 5% CO_2_ (72 hours before irradiation).

The irradiation was performed at the microbeam facility SNAKE^36^,^37^ at the Munich 14 MV tandem accelerator with one carbon ion (43 MeV, LET = 370 keV/μm) or 32 protons (20 MeV, LET = 2.6 keV/μm) per point in a 5 × 5 μm^2^ matrix, with an accuracy of 0.58 μm in x and 0.86 μm in y^35^ (see Fig. 1). One single 43 MeV carbon ion results in approximately 20 DSB along its approximately 7 μm track through the nucleus^8^, and the lowest number of protons necessary to obtain observable foci, which was about 30 protons per point, means an average number of three DSB that are distributed randomly along the ion track across the cell nucleus that has a height of about (7.2 ± 0.3) μm. During irradiation and the subsequent observation the cells were covered by cell culture medium and the temperature was kept constant at 37°C.

### Distance tracking with 2D Fluorescence Microscopy and quantitative dynamics analysis

Live cell observation started approximately 200–400 s after irradiation and time series of different interval length Δt (e.g. 5 s, 1 or 10 minutes) and total length t_total_ (20 minutes up to several hours) were recorded. Motion analysis was performed with the open source image analysis software ImageJ (http://rsb.info.nih.gov/ij) using the SpotTracker plugin (http://bigwww.ep.ch/sage/soft/spottracker/), developed by Daniel Sage et al^38^. for tracking fluorescent particles in dynamic image sequences with subpixel resolution. After visually verifying that the foci were tracked correctly, the two-dimensional distance l(t) of all neighboring foci pairs (as well as second next neighbors) were calculated from the x-y coordinates gathered by the SpotTracker algorithm and the standard deviation σ of the distance changes Δl_i_ was determined for different time intervals Δt for analysis of the foci dynamics. For more details see Supplementary Information.

## Author Contributions

S.G., V.H. and G.D. designed research; S.G., V.H., C.G., G.A.D. and C.S. performed research; S.G., V.H., M.H. and G.D. analyzed data; and S.G., V.H., G.D. and A.A.F. wrote the paper.

## Supplementary Material

Supplementary Information Supplementary Information
