# Supplementary material for: Subdiffusion Supports Joining Of Correct Ends During Repair Of DNA Double-Strand Breaks
Source: Sci Rep. 2013 Aug 27;3:2511. doi: 10.1038/srep02511 (PMC3753591; doi:10.1038/srep02511)
Supplement: Supplementary Information [file srep02511-s1.pdf]

SUBDIFFUSION SUPPORTS JOINING OF CORRECT ENDS DURING REPAIR OF DNA DOUBLE-STRAND BREAKS

S. Girst[1]*, V. Hable[1], G.A. Drexler[2], C. Greubel[1], C. Siebenwirth[1], M. Haum[1], A.A. Friedl[2], G. Dollinger[1]

[1]Angewandte Physik und Messtechnik LRT2, Universität der Bundeswehr München, 85577 Neubiberg, Germany.

[2]Department of Radiation Oncology, University of Munich, 80336 Munich, Germany.

Supplementary Information

Materials and Methods

Cell culture and irradiation

Generation of a stable transfectant (clone F1) of human osteosarcoma cells (U2OS) with pEGFP-MDC1 was described [34]. Twelve to 72 hours before irradiation the cells were seeded into custom-made cell containers [35] and cultivated in HEPES-buffered, phenol red-free medium supplemented with 0.25 mM Trolox at 37°C and 5 % CO_2.

The irradiation was performed at the microbeam facility SNAKE [36,37] at the Munich 14 MV tandem accelerator with one carbon ion (43 MeV, LET = 370 keV/µm) or 32 protons (20 MeV, LET = 2.6 keV/µm) per point in a 5x5 μm^2 matrix, with an accuracy of 0.58 µm in *x* and 0.86 µm in *y* [35] (see Fig. 1). In some experiments, 4x4 μm^2 or 6x6 μm^2 matrices were used. The initial energy of the carbon ions was 55 MeV, which was reduced to 43 MeV at the position of the cells. The energy reduction is caused by the vacuum exit window (7.5 µm Kapton), the entrance window of the cell container (5 µm Mylar) and about 30 µm of growth medium that the ions have to traverse to reach the cells. The energy of the protons is not much influenced by this stack of materials. One single 43 MeV carbon ion results in approximately 20 double-strand breaks along its approximately 7 µm track through the

nucleus [8], and the lowest number of protons necessary to obtain observable foci, which was about 30 protons per point, means an average number of three double-strand breaks that are distributed randomly along the ion track across the cell nucleus that has a height of about (7.2 ± 0.3) µm. During irradiation and the subsequent observation the cells were covered by cell culture medium and the temperature was kept constant at 37°C.

Distance tracking with 2D Fluorescence Microscopy

Microscopic 2D online observation of MDC1 foci began 200-400 s after irradiation as soon as the matrix pattern of the GFP-tagged repair protein foci was visible with a sufficiently high signal-to-noise ratio in the epifluorescence microscope (Zeiss Axiovert 200M, objective Zeiss Plan-Apochromat 40x/0.95 Korr Ph3), which is tilted by 90° and is part of the SNAKE target station. 20 % - 40 % of the irradiated cells expressed a suitable amount of GFP-tagged molecules to be quantitatively analyzed. With the “Smart Experiments” tool of the Zeiss AxioVision software time series of different interval length Δt (e.g. 5 s, 1 or 10 minutes) and total length t_{total} (20 minutes up to several hours) were recorded by a Zeiss AxioCam MRm CCD camera, enabling the observation and tracking of foci pairs for many different time intervals Δt. Images were taken mostly at 60 s or 600 s intervals. In some experiments, also 5 s and 180 s intervals were used.

To allow long observation times with a sufficient number of images, illumination was performed by a commercially available LED light source for fluorescence microscopy (Zeiss Colibri), which reduces photobleaching and phototoxicity effects significantly as compared to a mercury arc lamp, since no UV light is emitted.

Motion analysis was performed with the open source image analysis software ImageJ (http://rsb.info.nih.gov/ij) using the SpotTracker plugin (http://bigwww.ep.ch/sage/soft/spottracker/), developed by Daniel Sage et al. [38] for tracking fluorescent particles in dynamic image sequences with subpixel resolution.

Quantitative dynamics analysis

Motion analysis includes Mexican-hat filtering (called “SpotEnhancingFilter” in the plugin), which is optimally tailored for the detection of a Gaussian-like spot in noisy images [38], and a tracking algorithm that uses dynamic programming to extract the optimal (*x,y,t*) trajectory of one particle at a time. This is done by optimizing a cost function, which weights in a user-defined way the intensity of the manually chosen spot, its variation from one frame to the next and the movement, which can be restricted to a maximal displacement (e.g. 10 pixels, or more for fast-moving cells). In this work we set the weighting parameters “intensity factor” and “movement constraint” to 20 %, “intensity variation” to 80 % and “centre constraint” to 0%.

After visually verifying that the foci were tracked correctly, the two-dimensional distance $l(t)$ of all neighbouring foci pairs were calculated from the *x-y* coordinates gathered by the SpotTracker algorithm. We take the standard deviation $\sigma^2(\Delta l(\Delta t))$ of the changes in foci distances $\Delta l_i(\Delta t)$ as a measure for the underlying random walk process instead of the mean squared distance change $<\Delta l^2(\Delta t)> = \sigma^2(\Delta l(\Delta t)) + L^2$ (eq. (S5)) as used by [18,19] and others, in order to make the measurement insensitive to a possible growth of the cell nucleus that contributes to an average distance change $L = <\Delta l(\Delta t)>$ of the foci. We measured an average growth of L of about 1% (see below) which means an average 100 nm increase of the initially 10 µm diameter of the cell nuclei in 2 hours.

The standard deviation $\sigma^2(\Delta t)$ of the $\Delta l_i(\Delta t)$ is twice the mean square displacement $MSD(\Delta t)$ by random walk of a single particle after a time interval Δt. Using eq. (1) one obtains:

$$\sigma^2(\Delta t) \equiv \sigma^2(\Delta l(\Delta t)) = 2MSD(\Delta t) = \frac{4dD_\alpha}{\Gamma(1+\alpha)}\Delta t^\alpha \qquad \text{eq. (S1)}$$

with the dimensionality being d = 2 for our case. The standard deviation σ of the distance changes Δl_i (cf. Fig. S1), which reveals the underlying foci dynamics, is independent of directional motion (“drift”) of foci in the nucleus (e.g. due to cell deformation) and therefore a better measure for the foci dynamics than the mean of the squared distance changes Δl_i^2. The two quantities are equivalent in

the case of no additional drift and are twice the mean square displacement MSD of a single particle (eq. (S1)). For every evaluable cell $\sigma(\Delta t)$ was calculated from the Δl_i of neighbouring foci (separately for vertical and horizontal pairs) and then averaged over all analyzed cells. For comparison second next neighbours distances were analyzed as well. Each cell sample consisted of 4 different, independently performed experiments. The various experiments did not differ within the given accuracy and thus the data have been pooled. The measurement uncertainty σ_0 (estimated to approximately 100 nm from the precision of the position of each focus centre) was quadratically subtracted from the measured σ^2.

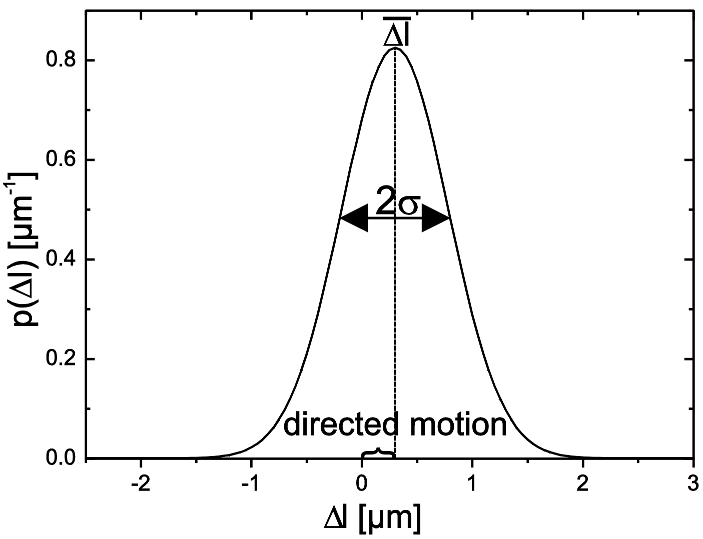

Fig. S1 Distribution function $p(\Delta l,\Delta t)$ of the distance changes Δl for normal diffusion. The mean $\overline{\Delta l}$ of the Gaussian distribution is shifted to a positive value which indicates a directed motion, e.g. a deformation acting on the cell. The standard deviation σ of the distribution is a measure for the underlying diffusion.

Comparison of subdiffusion and constrained diffusion modelling

The diffusion data obtained from the distance analysis after carbon irradiation was also fitted with a model of constrained diffusion as proposed by Jegou et al.[19]. The equation describing a confined diffusion in 2D with a diffusion coefficient D_c in a region of radius r_c is [19]

$$\sigma^2(\Delta t) = 2\left\langle r_c^2 \right\rangle \cdot \left[1 - \exp\left(-\frac{4 D_c \Delta t}{\left\langle r_c^2 \right\rangle} \right) \right] \qquad \text{eq. (S2)}$$

when both foci diffuse with the same D_c in the same radius r_c. Assuming an additional normal diffusion of this region with a diffusion constant D_n leads to [19]

$$\sigma^2(\Delta t) = 2\left\langle r_c^2 \right\rangle \cdot \left[1 - \exp\left(-\frac{4 D_c \Delta t}{\left\langle r_c^2 \right\rangle} \right) \right] \cdot \left(1 + \frac{4 D_n \Delta t}{\left\langle r_c^2 \right\rangle} \right) \qquad \text{eq. (S3)}$$

A comparison of these two models with the subdiffusion model is displayed in fig. S2. The linear (fig. S2*a*) and double-logarithmic (fig. S2*b*) plots of these fits demonstrate that the constrained diffusion models do not fit the experimental data (R^2 = 0.91 and reduced χ^2 = 5.0 for the constrained diffusion model and R^2 = 0.95 and reduced χ^2 = 2.8 for the constrained plus normal diffusion model). Furthermore, these models yielded different results for the confinement radius after proton and carbon irradiation ((346 ± 18) nm vs. (216± 9) nm), which is biologically implausible.

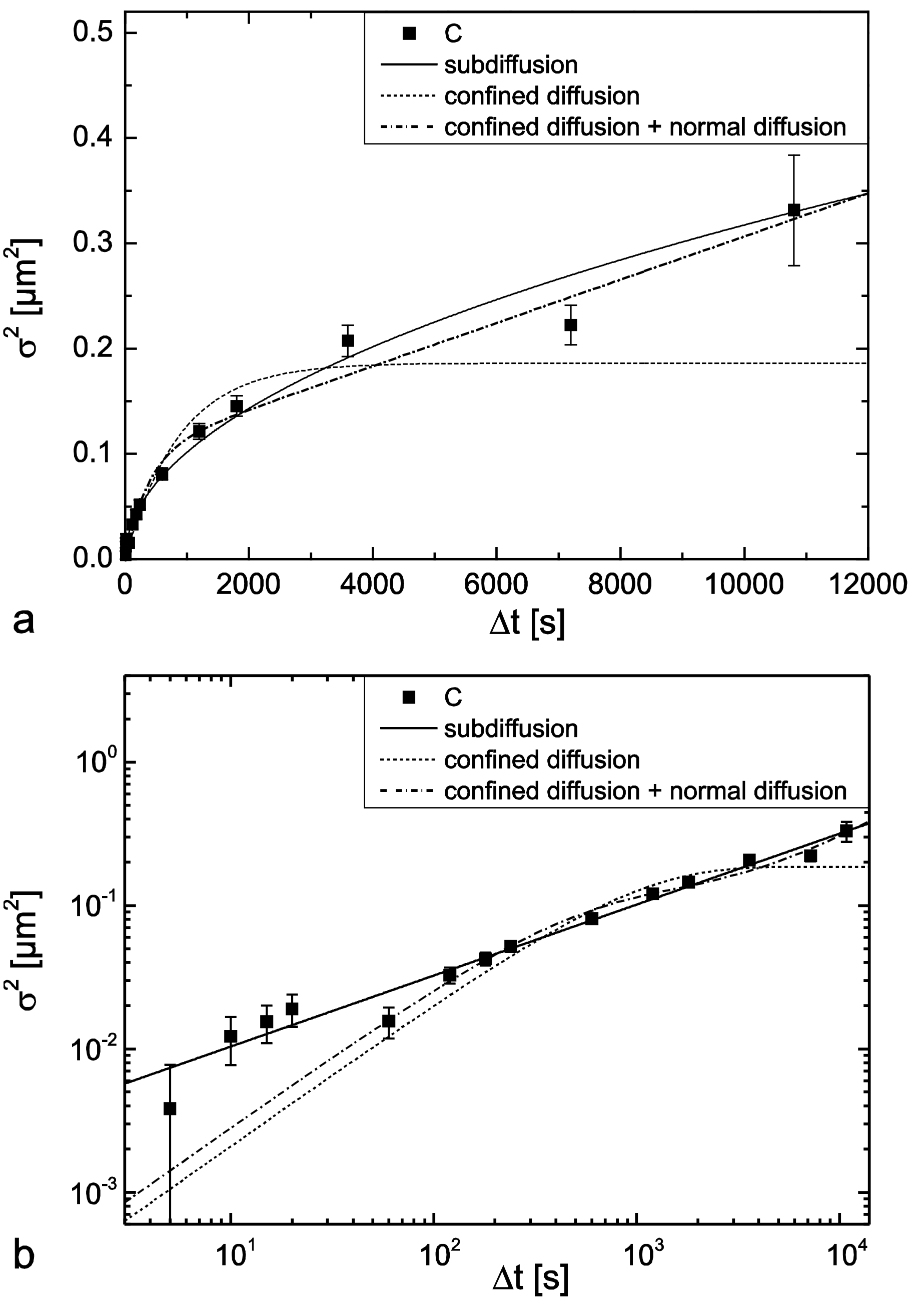

Fig. S2. Linear (*a*) and double-logarithmic (*b*) plot of the squared standard deviations σ^2 (± SEM) of the distance changes $\Delta l(\Delta t)$ between neighbouring MDC1 foci in the nuclei of cells irradiated with carbon ions . The data are fitted with the power-law function for subdiffusion (eq. (2)), with eq. (S2) for constrained diffusion and with eq. (S3) for constrained diffusion plus normal diffusion of the region of constrain. The confinement radius r_c in the constrained diffusion model is (216± 9) nm, and (225± 10) nm in the model of additional normal diffusion of the confinement region. The diffusion coefficient D_c is (2.6± 0.2) x 10^{-5} µm^2/s and (3.6± 0.3) x 10^{-5} µm^2/s, respectively and the normal diffusion constant D_n in the third model is (2.6± 0.4) x 10^{-6} µm^2/s.

Comparison of mobility after different post-irradiation intervals

In one sample, time series with time lag Δt = 60 s were recorded directly after irradiation and one hour later (from 60-80 min after irradiation) (see Fig. S3), showing a slightly slower but not significant change in mobility. Thus ergodicity is well represented for the underlying subdiffusion process.

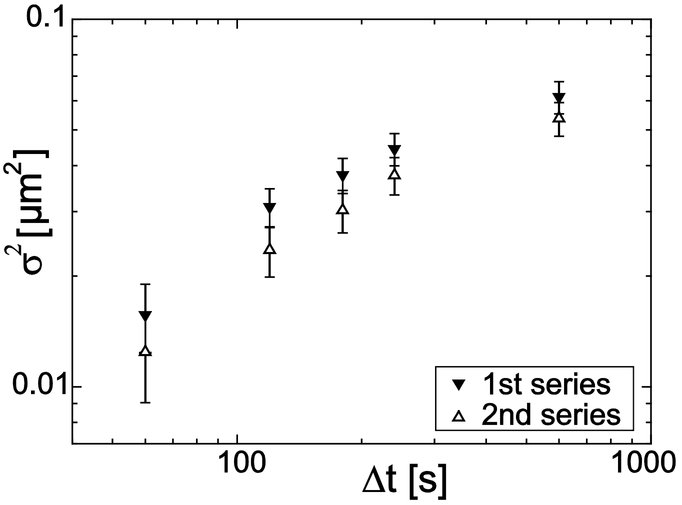

Fig. S3. Comparison of mobility determined from time-series recorded directly after irradiation ("1st series", first 20 min) and from later times ("2nd series", 80-100 min after irradiation) evaluated in the same sample.

Cell cycle dependence

As the cells had not been synchronized according to their cell cycle phase, irradiation took place in all phases. Thus the $\sigma^2(\Delta t)$ values extracted are the average of all cells evaluated. However, a few percent of the cells could not be included into the analysis because they showed a very different behaviour of the foci dynamics which did not allow the extraction of the movements in the same way as presented before. These cells were coincidently irradiated after the checkpoint for mitosis, so that these cells started dividing even despite the irradiation. A series of pictures that includes one of these cells dividing during the analysis and some others that do not divide and behave as usual are shown in Fig. S4. Even through the cell division process certain MDC1-foci can be observed. The daughter cells also showed repair protein foci, often in a similar pattern to that irradiated in the mother cell (see Fig. S4, 1:00:45 h after irradiation).

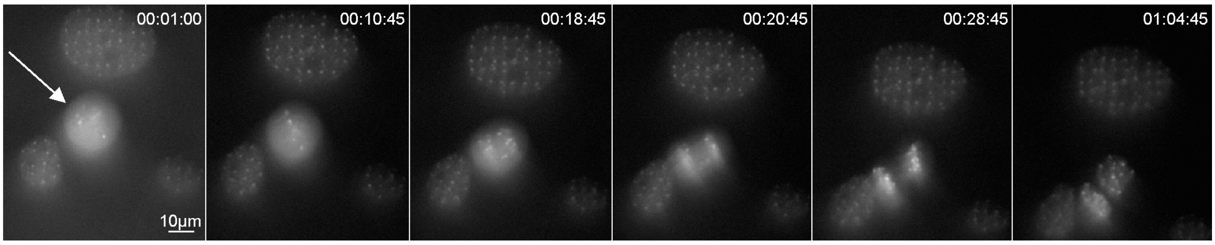

Fig. S4. Time series of U2OS cells irradiated with carbon ions in a 4x4 μm^2 matrix. The arrow indicates a cell undergoing mitosis despite irradiation, starting about 20 minutes after irradiation. The other cells clearly show the matrix pattern during the complete observation time (1 h). Time indication hours:minutes:seconds (hh:mm:ss). A similar foci pattern as in the undivided cells can be recognized in the divided cell.

Influence of changes of the nucleus size after irradiation

Analyzing the mean squared distance change $<\Delta l^2(t)>$ of IRIF pairs during a time interval t after irradiation is a way of determining the random walk behaviour of the individual IRIF. The ensemble-averaged $<\Delta l^2(t)>$ of N IRIF pairs with an initial distance l_0 (at the time of the irradiation) is calculated according to

$$\left\langle \Delta l^2(t)\right\rangle = \frac{1}{N}\sum_{i=1}^{N}\left(l_i(t)-l_{i0}\right)^2 = \left\langle l^2(t)\right\rangle - 2l_0\left\langle l(t)\right\rangle + l_0^2 = \left\langle l^2(t)\right\rangle - 2l_0\left\langle l_0+\Delta l(t)\right\rangle + l_0^2 = \left\langle l^2(t)\right\rangle - l_0^2 - 2l_0\left\langle \Delta l(t)\right\rangle \quad \text{eq. (S4)}$$

Adapting

$$\left\langle l^2(t)\right\rangle = l_0^2 + 2MSD(t) = l_0^2 + \sigma^2(t) \quad \text{eq. (S5)}$$

which describes the squared distance l^2 after a random walk process for a change in l_0 due to a change in nucleus size $L(t)$ in the time interval t, yields

$$\left\langle l^2(t)\right\rangle = \left(l_0 + L(t)\right)^2 + \sigma^2(t) = l_0^2 + \sigma^2(t) + 2l_0L(t) + L(t)^2 \quad \text{eq. (S6)}$$

Introducing this into the equation for the mean squared distance change (eq. (S4)) gives

$$\left\langle \Delta l^2(t)\right\rangle = l_0^2 + \sigma^2(t) + 2l_0L(t) + L(t)^2 - l_0^2 - 2l_0\left\langle \Delta l(t)\right\rangle = \sigma^2(t) + L(t)^2 + 2l_0\left(L(t) - \left\langle \Delta l(t)\right\rangle\right)$$
$$= \sigma^2(t) + L(t)^2 \quad \text{eq. (S7)}$$

The last equivalence occurs because the mean distance change only due to random approaches 0 for large values of N and thus the mean of the distance changes of all IRIF pairs during t is equal to the change of the nucleus size, $<\Delta l(t)>=L$.

This means that measuring the mean squared distance change $<\Delta l^2(t)>$ of IRIF can overestimate the real random walk behaviour in case of a change of nucleus size during the time interval t.

The ensemble average of the $l_i^2(t)$ (i.e. $<l^2(t)>$) for different time points t after irradiation reveals this average distance change of the foci pairs $L(t) = <\Delta l(t)>$ with time and thus whether the cell nuclei change their sizes after irradiation (see eq. (S4)). We see an increase of $<l^2(t)>$ that is larger than the contribution from the increase of $\sigma^2(t)$ (see Fig. S5). The additional contribution reflects an average

growth of the foci distances L/l_0 ~ 1-2% within 2 hours that corresponds to a growth of the diameter of the cell nucleus of 100-200 nm for a 10 µm sized nucleus.

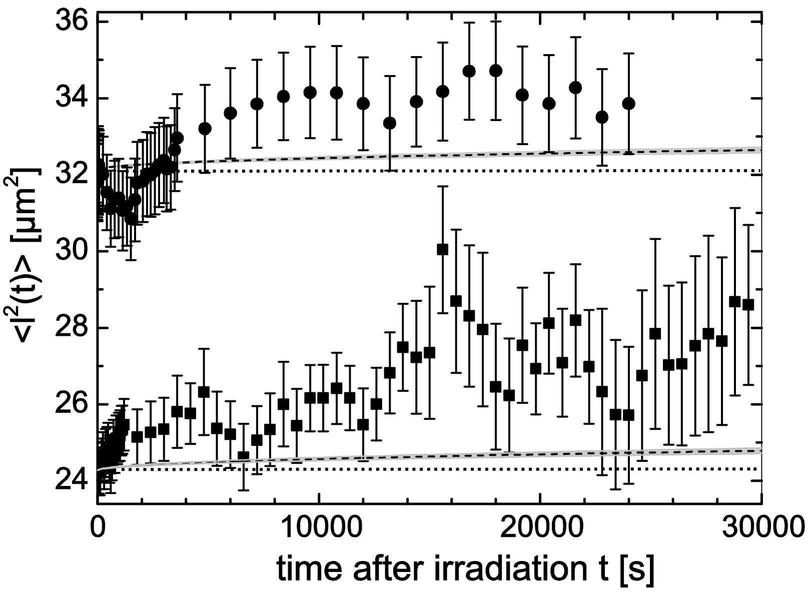

Fig. S5. Mean squared distance $<l^2>$ of all foci pairs with initial distance $<l_0>$ ~ 4.9 µm (squares) and $<l_0>$ ~ 5.7 µm (circles) as a function of time after irradiation *t*. For each sample, the mean squared initial distance $<l_0^2>$ (dotted line) and the contribution of a random walk with σ^2 (dashed line, with error "bars" in grey)) are given for comparison. Error bars for $<l_0>$ are dominated by the accuracy of the matrix application.

Random walk of the centre of mass of N DSB

The time-averaged MSD of 1 DSB (averaged over the *M* time points t_j of a time series) is defined as [39]

$$MSD_1(\Delta t) = \frac{1}{M}\sum_{j=1}^{M}\left[\vec{r}\left(t_j + \Delta t\right) - \vec{r}\left(t_j\right)\right]^2$$

Therefore the time-averaged MSD of the centre of mass of *N* DSB (at positions $\vec{r}_i$) is given by

$$MSD_{CM}(\Delta t) = \frac{1}{M}\sum_{j=1}^{M}\left[\vec{r}_{CM}\left(t_j + \Delta t\right) - \vec{r}_{CM}\left(t_j\right)\right]^2 = \frac{1}{M}\sum_{j=1}^{M}\left[\frac{1}{N}\sum_{i=1}^{N}\vec{r}_i\left(t_j + \Delta t\right) - \frac{1}{N}\sum_{i=1}^{N}\vec{r}_i\left(t_j\right)\right]^2$$

$$= \frac{1}{M}\sum_{j=1}^{M}\frac{1}{N^2}\left[\sum_{i=1}^{N}\vec{r}_i\left(t_j + \Delta t\right) - \vec{r}_i\left(t_j\right)\right]^2$$

$$= \frac{1}{N^2}\sum_{i=1}^{N}\left(\frac{1}{M}\sum_{j=1}^{M}\left[\vec{r}_i\left(t_j + \Delta t\right) - \vec{r}_i\left(t_j\right)\right]^2\right) - \frac{2}{N^2 M}\sum_{j=1}^{M}\sum_{i=1}^{N}\sum_{k=i+1}^{N}\left(\left[\vec{r}_i\left(t_j + \Delta t\right) - \vec{r}_i\left(t_j\right)\right]\cdot\left[\vec{r}_k\left(t_j + \Delta t\right) - \vec{r}_k\left(t_j\right)\right]\right)$$

$$= \frac{1}{N^2}\sum_{i=1}^{N}MSD_{1,i}(\Delta t) - \frac{2}{N^2 M}\sum_{j=1}^{M}\sum_{i=1}^{N}\sum_{k=i+1}^{N}\Delta\vec{r}_i\left(t_j, \Delta t\right)\cdot\Delta\vec{r}_k\left(t_j, \Delta t\right) = \frac{1}{N}MSD_1(\Delta t) - \ldots$$

$$\cong \frac{1}{N}MSD_1(\Delta t)$$

since the last term is small compared to the first term for large numbers of time points *M* (as the dot product reaches random negative and positive values). This means that the MSD (and thus also σ^2) of the centre of mass of *N* DSB appears to be smaller than the random walk behaviour of a single DSB by a factor 1/*N*.

References (as in Article)

8. Hauptner, A. *et al.* Spatial Distribution of DNA Double-Strand Breaks from Ion Tracks, *Royal Danish Academy of Sciences and Letters, Copenhagen,* 59–85 (2006b).

19. Jegou, T. *et al.* Dynamics of telomeres and promyelocytic leukemia nuclear bodies in a telomerase-negative human cell line, *Mol. Biol. Cell* **20,** 2070–2082 (2009).

34. Hable, V. *et al.* Recruitment Kinetics of DNA repair proteins Mdc1 and Rad52 but not 53BP1 depend on damage complexity, *PLoS ONE* **7,** e41943 (2012).

35. Hable, V. *et al.* The live cell irradiation and observation setup at SNAKE, *Nuclear Instruments and Methods in Physics Research Section B: Beam Interactions with Materials and Atoms* **267,** 2090–2097 (2009).

36. Datzmann, G. *et al.* The Munich microprobe SNAKE: First results using 20 MeV protons and 90 MeV sulfur ions. 7th International Conference on Nuclear Microprobe Technology and Applications, *Nuclear Instruments and Methods in Physics Research Section B: Beam Interactions with Materials and Atoms* **181,** 20–26 (2001).

37. Hauptner, A. *et al.* Microirradiation of cells with energetic heavy ions, *Radiat Environ Biophys* **42,** 237–245 (2004).

38. Sage, D. Neumann, F. R. Hediger, F. Gasser, S. M. & Unser, M. Automatic tracking of individual fluorescence particles: application to the study of chromosome dynamics, *IEEE Trans Image Process* **14,** 1372–1383 (2005).

39. Burov, S. Jeon, J.-H. Metzler, R. & Barkai, E. Single particle tracking in systems showing anomalous diffusion: the role of weak ergodicity breaking, *Phys Chem Chem Phys* **13,** 1800–1812 (2011).
